# Predictive and Prognostic Value of Metabolic Tumor Volume (MTV) in Patients with Laryngeal Carcinoma Treated by Radiotherapy (RT) / Concurrent Chemoradiotherapy (CCRT)

**DOI:** 10.1371/journal.pone.0117924

**Published:** 2015-02-18

**Authors:** Kenichiro Yabuki, Osamu Shiono, Masanori Komatsu, Daisuke Sano, Goshi Nishimura, Masahiro Takahashi, Takahide Taguchi, Tomio Inoue, Nobuhiko Oridate

**Affiliations:** 1 Department of Otorhinolaryngology and Head and Neck Surgery, Yokohama City University School of Medicine, Yokohama, Japan; 2 Department of Radiology, Yokohama City University School of Medicine, Yokohama, Japan; INRS, CANADA

## Abstract

**Purpose:**

To evaluate the predictive and prognostic value of pretreatment metabolic tumor volume (MTV) in patients with treated by radiotherapy (RT) or concurrent chemoradiotherapy (CCRT).

**Methods:**

We reviewed the records of 118 patients with newly diagnosed laryngeal carcinoma, who had been treated by RT or CCRT. Pretreatment positron emission tomography (PET) was performed, and MTV values were obtained by contouring margins of standardized uptake value. Clinical factors and MTV were analyzed for their association with survival.

**Results:**

Patients with residual disease showed a significantly higher MTV than those with a complete response (CR) after primary treatment. Univariate analysis showed that the patients with a high MTV had a significantly lower disease-free survival (DFS) (*p* < 0.001). Subsite (*p* = 0.010), T-stage (*p* < 0.001), nodal metastasis (*p* < 0.001) and clinical stage (*p* < 0.001) also correlated significantly with DFS. In the multivariate analysis, MTV and clinical stage were both found to be independent prognostic factors for DFS (*p* = 0.001, *p* = 0.034, respectively). The 3-year DFS for patients with a high MTV were significantly poorer than those with a low MTV (*p* < 0.001).

**Conclusions:**

MTV of the primary tumor is a significant prognostic factor for DFS in patients with laryngeal carcinoma treated by RT or CCRT. The results imply that MTV could be an important factor when planning treatment and follow-up for patients with laryngeal carcinoma.

## Introduction

Surgery and subsequent radiation therapy (RT) has become the treatment of choice for early and locally advanced head and neck cancer. In patients with laryngeal cancer, phonetic function is sacrificed when surgical resection is performed. RT is, therefore, used for the purpose of functional preservation, and concurrent chemotherapy is added with the intention of controlling micrometastasis and/or to act as a radiosensitizers [1]. Patients sometimes experience a residual or recurrent tumor after RT or concurrent chemoradiotherapy (CCRT), and salvage surgery for these patients can result in an increased risk of perioperative complications. For this reason, precise prediction of the response to RT or CCRT is important when selecting the optimal treatment strategy. The Union Internationale Contre le Cancer (UICC) staging system or the American Joint Committee on Cancer (AJCC) staging system is generally applied for staging. However, the prognostic value of these staging systems is limited as they are based on tumor morphology, not on individual biological and molecular characteristics [2].

The efficacy of ^18^F labeled fluoro-deoxyglucose (FDG) positron emission tomography (PET) and fusion images of PET with computed tomography (PET/CT) have been reported [3], with the authors concluding that PET should be used routinely in combination with CT or magnetic resonance imaging (MRI) to improve nodal or distant-disease staging and to detect the recurrence of head and neck cancer. Maximum standardized uptake value (SUVmax) is the most extensively studied FDG parameter in head and neck squamous carcinoma (HNSCC) [2]. However, there is growing interest in FDG volumetric parameters, such as metabolic tumor volume (MTV), which could be superior to SUVmax as predictive parameters [4–8]. The fact that MTV measures tumor volume, thus reflecting the metabolic activity of the whole tumor, whereas SUVmax only provides information on a single point within the tumor, is considered to result in differences in their respective prognostic values [4].

The clinical behavior of HNSCCs varies according to the primary site. The prognostic value of MTV in HNSCCs including several sites has been reported [4–8]. We, therefore, focused on MTV during pretreatment PET or PET/CT examinations in patients with laryngeal cancer. In this study, the predictive value of pretreatment MTV in relation to clinical response after radiotherapy alone or with concurrent chemotherapy was assessed. Its prognostic significance was also investigated by estimating disease-free survival according to MTV status.

## Materials and Methods

### Patients

Between March 2005 and April 2012, 139 patients with squamous cell carcinoma of the larynx were diagnosed and treated in the Department of Otorhinolaryngology, Head and Neck Surgery, at Yokohama City University Hospital, Japan. Patients underwent diagnostic procedures which included: medical history; physical examination; CT imaging from the skull base to the diaphragm; panendoscopy (nasopharyngoscopy, laryngoscopy and esophagogastroscopy); histological examination of the larynx; and PET or PET/CT imaging. PET imaging was performed within 4 weeks before the start of irradiation. Ten patients were excluded due to an absence of or inadequate PET data for further analysis.

A treatment plan was formulated on the basis the TNM classification of the 2002 Union Internationale Contre le Cancer (UICC) staging system. Eleven patients underwent primary surgical resection, with the remaining 118 patients eligible for this study. RT was delivered 5 days a week using a single daily fraction of 1.8 or 2.0 Gray (Gy). The median radiation dose for patients enrolled in the study was 70.0Gy (range 27.0–70.2Gy). CCRT was performed in 110 cases. We applied a high-dose chemotherapy regimen to the patients with stage III/IV tumors without comorbidity and who were aged of 75 years or younger [9]. This high-dose regimen, which was applied to 26 patients (22.0%), consisted of either a TPF regimen (docetaxel (50 mg/m^2^, Day 1 and 29), cisplatin (60 mg/m^2^, Day 4 and 33), and 5-fluorouracil (5-FU) (600 mg/m^2^ given over 24 h for 5 days, Days 1–5 and 29–34)) or a PFML (modified PF) regimen (cisplatin (60 mg/m^2^, Day 4 and 33), 5-FU (600 mg/m^2^ given over 24 h for 5 days, Days 1–5 and 29–34), methotrexate (30 mg/m^2^, Day 1 and 29), and leucovorin (20 mg/m^2,^ Days 1–5 and 29–34).

A low-dose chemotherapy regimen consisted of either a CBDCA-UFT regimen (carboplatin (CBDCA) at a dose of the area under the curve (AUC) of 1.25 weekly and oral uracil-tegafur (UFT, 300mg daily)), an S-1 regimen (oral tegafur at 40 or 50 mg twice a day or 2 weeks followed by 1 week of rest), or a weekly docetaxel regimen (12 mg/m^2^/week). The low dose regimen was selected for the patients with renal dysfunction (24-h creatinine clearance < 60 mL/min), history of ischemic heart disease, brain infarction, or peptic ulcer, or those older than 75 years of age or having stage I/II tumor [9]. The low-dose regimen was applied to 73 patients (61.9%). For patients in whom the application of these regimens was considered likely to result in severe toxicity, thereby interrupting irradiation, RT with oral UFT (11 patients, 9.3%) or RT alone (8 patients, 6.8%) was performed.

After treatment, initial tumor responses were evaluated according to RECIST criteria [10] at 2 months after completion of RT or CCRT. Follow-up nasopharyngoscopy and laryngoscopy were performed at least every three months, and radiological examinations, such as CT and/or PET, were repeated at least every six months in the all surviving patients. In cases in which recurrence was suspected, the examination and treatment plan was designed accordingly. Written informed consent to participate in this study as well as future statistical studies was received from all patients prior to the start of the diagnostic procedures.

### PET and MTV measurements

Patients were asked to fast for at least 5 h and their blood sugar levels were checked before FDG injection. Images were acquired from the top of the head to the mid-thigh at 60 min after the intravenous injection of 2.5–5.0 MBq/kg of FDG. Scans were acquired with a PET scanner (SET 2400, Shimadzu, Kyoto, Japan) or a PET/CT device (Aquiduo 16, Toshiba Medical Systems, Tokyo, Japan). The capacity of resolution of these two systems were 4 mm and 2 mm, respectively. And cross calibration was performed so that two systems could derive identical SUV. Interpretation of PET images was based on a consensus between two experienced radiologists regarding the focal accumulation of FDG at a higher level than that of the surrounding background tissue. PET/CT was introduced in our institution in 2007. The patients underwent PET alone were also performed corresponding CT of the neck within intervals of 15 days.

MTV values were computed from attenuation-corrected PET data using a commercial software package (SYNAPSE VINCENT, Fujifilm medical Co., Tokyo, Japan) included in the electric medical chart system in our hospital. First, PET data were read into the workstation in DICOM format, and accumulation of FDG was reconstructed in three dimensions. Then, using a graphical interface, a volume of interest was drawn to enclose the whole hypermetabolic tumor lesion. The primary tumor volume was delineated by applying standardized uptake value (SUV) = 2.0, 2.5, and 3.0 isocontours. These volumes were defined as MTV _(T, 2.0)_, MTV _(T, 2.5)_, MTV _(T, 3.0)_, respectively. In the patients with neck lymph node metastasis, the volume of the tumor plus that of metastatic lymph nodes was also measured. In the patients with bilateral neck lymph node metastasis, nodal MTV was summated bilaterally. These volumes were defined as MTV _(T+N, 2.0)_, MTV _(T+N, 2.5)_, MTV _(T+N, 3.0)_, respectively. This study was approved by the Internal Review Board of Yokohama City University Hospital.

### Statistical analysis

Statistical differences in predictive values were determined by Mann-Whitney’s U test. Time-to-event statistics were calculated from the first day of irradiation to the event of interest. Locoregional control was defined as the time to the first locoregional recurrence. Disease-free survival (DFS) was defined as the time to the first relapse of the disease or death from any reason. Overall survival (OS) and DFS were calculated using the Kaplan–Meier method and comparison between the groups was carried out using the Wilcoxon log rank test. Logistic regression analysis was used for univariate comparisons, and multivariate logistic regression analysis was used as multivariate statistical technique. Statistical analysis was performed with EZR (Saitama Medical Center, Jichi Medical University), which is a graphical user interface for R (The R Foundation for Statistical Computing, Vienna, Austria). Statistical tests were two-sided and *p* values <0.05 were considered significant.

## Results

### Patient characteristics

Patient characteristics are summarized in Table 1. Of the 118 patients enrolled, 115 (97.5%) were male and three were female, and their median age was 68 (range 48–89, mean 68.1) years. Eighty-four (71.2%) patients had a tumor in glottic region, 31 (26.3%) in the supraglottic region and 3 (2.5%) in the subglottic region. Twenty-eight patients (23.7%) were diagnosed as having lymph node metastasis. Of 28 patients with nodal metastasis, 15 were unilateral and 13 were bilateral. With regard to tumor stage, 49 (41.5%) patients presented with a stage II tumor, 29 (24.6%) with a stage IV tumor, 25 (21.2%) with a stage III tumor, and 15 (12.7%) with a stage I tumor.

**Table 1:** Patient characteristics (n = 118).

Characteristics	No.	%
**Age (years)**		
Median	68	
Range	48–89	
**Gender**		
Male	115	97.5
Female	3	2.5
**Subsite**		
Glottic	84	71.2
Supraglottic	31	26.3
Subglottic	3	2.5
**cT stage**		
T1	16	13.6
T2	57	48.3
T3	31	26.3
T4	14	11.9
**cN stage**		
N0	90	76.3
N1	5	4.2
N2	21	17.8
N3	2	1.7
**TMN stage**		
I	15	12.7
II	49	41.5
III	25	21.2
IV	29	24.6
**Treatment modality**		
Radiotherapy alone	8	6.8
CCRT	110	93.2
High dose regimen	(26)	(22.0)
Low dose regimen	(73)	(61.9)
Oral UFT	(11)	(9.3)
**Treatment response**		
CR	100	84.7
non-CR	18	15.3
**Recurrence after CR**	11	9.3
**Follow up duration, months**		
Median	36	
Range	3–100	

Abbreviations: CCRT = concurrent chemoradiotherapy; UFT = uracil-tegafur; CR = complete response.

### MTV cut-off values

**Fig 1:**
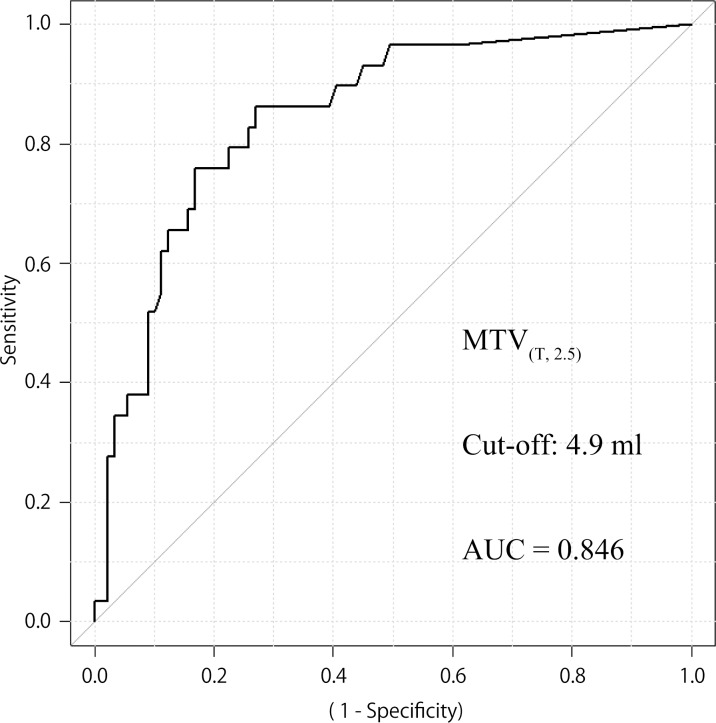
ROC curves for patients with locoregional control according to MTV _(T, 2.5)_ status. AUC 0.846 (95% CI 0.766–0.925, *p* < 0.001), cut off value 4.9 ml for MTV _(T, 2.5)_ (n = 118). Sensitivity and specificity of the dichotomized MTV _(T, 2.5)_ (≥ 4.9 vs < 4.9) were 0.759 and 0.831, respectively.

**Fig 2:**
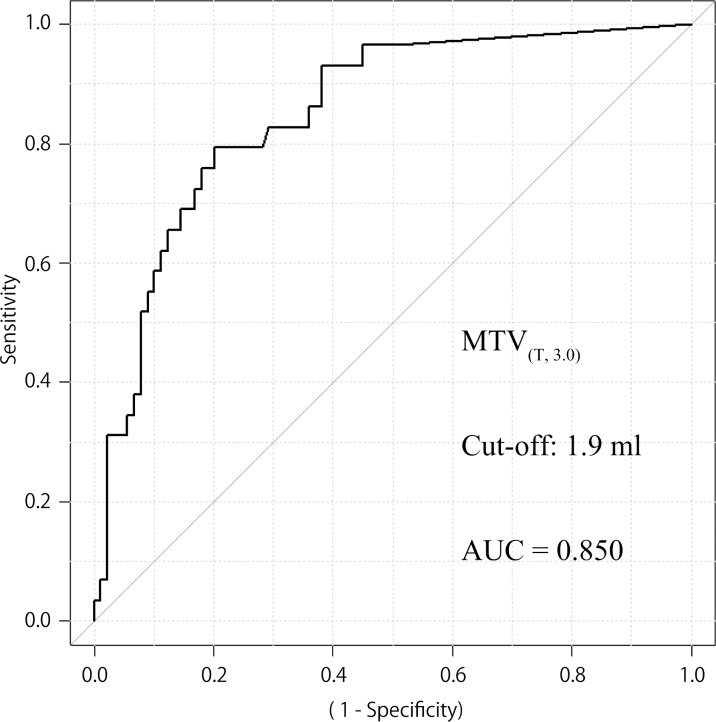
ROC curves for patients with locoregional control according to MTV _(T, 3.0)_ status. AUC 0.850 (95% CI 0.774–0.927, *p* < 0.001), cut off value 1.9 ml for MTV _(T, 3.0)_ (n = 118). Sensitivity and specificity of the dichotomized MTV _(T, 3.0)_ (≥ 1.9 vs < 1.9) were 0.793 and 0.798, respectively.

The median MTV _(T, 2.0)_, MTV _(T, 2.5)_, MTV _(T, 3.0)_ were 3.7 ml, 1.3 ml, 0.5 ml, and the median MTV _(T+N, 2.0)_, MTV _(T+N, 2.5)_, MTV _(T+N, 3.0)_ were 38.5 ml, 25.0 ml, 16.9 ml, respectively. A receiver operating characteristic (ROC) curve was used to estimate the MTV cut-off point for discriminating DFS. The optimal cut-off point was defined as the point nearest the upper-left corner of the chart. As a result, cut-offs of 6.3 ml, 4.9 ml, 1.9 ml were calculated to be significant for MTV _(T, 2.0)_, MTV _(T, 2.5)_, MTV _(T, 3.0)_, with the areas under the curve being 0.832, 0.846 (Fig. 1 (95% CI 0.766–0.925, *p* < 0.001)), 0.850 (Fig. 2 (95% CI 0.774–0.927, *p* < 0.001)), respectively. Cut offs of 36.4 ml, 19.6 ml, 18.1 ml were calculated to be significant for MTV _(T+N, 2.0)_, MTV _(T+N, 2.5)_, MTV _(T+N, 3.0)_, with the areas under the curve being 0.646, 0.683, 0.679, respectively.

### Treatment outcome and follow-up

A complete response (CR) after CCRT or RT was confirmed histologically or clinically in 100 patients, with residual lesions observed in 18 patients. The mean MTV _(T, 2.0)_ values in the 100 patients with a CR and in the 18 patients with residual disease were 7.3 ml and 31.6 ml, respectively (*p* < 0.001). In addition, the mean MTV _(T, 2.5)_ values were 4.5 ml and 22.3 ml (*p* < 0.001), and the mean MTV _(T, 3.0)_ values were 3.1 ml and 16.3 ml, respectively (*p* < 0.001). In the existence of a primary tumor with low MTV value (MTV _(T, 2.5)_ < 4.9 ml), sensitivity, specificity, accuracy, positive predictive value (PPV) and negative predictive value (NPV) for a CR after primary treatment were 0.79, 0.89, 0.81, 0.98, and 0.43, respectively.

Recurrence during follow-up was detected in 11 (11%) of 100 patients with a CR after primary treatment. Local recurrence was observed in five patients and locoregional recurrence was occurred in three patients. Distant metastasis was found in three patients during follow-up period. Six (28.6%) of 21 CR patients with a high MTV (MTV _(T, 2.5)_ ≥ 4.9 ml) and five (6.3%) of 79 CR patients with a low MTV (MTV _(T, 2.5)_ < 4.9 ml) experienced tumor recurrence. The CR patients with a high MTV showed a significant risk of recurrence even if the primary RT or CCRT resulted in a CR (*p* = 0.004, Chi square test).

Salvage total laryngectomy was performed in nine patients. At the final follow-up, 104 patients were alive and 14 had died: nine from laryngeal cancer and five from other causes. The median follow-up for surviving patients was 36 months.

### Patient survival according to MTV status

We next compared DFS and OS by clinical variable and MTV _(T, 2.5)_ using univariate and multivariate analyses (Tables 2 and 3). Univariate analysis showed that the patients with an MTV _(T, 2.5)_ ≥ 4.9 ml had a significantly lower DFS and OS *(p* < 0.001, *p* = 0.001). Subsite (*p* = 0.010, *p* = 0.024), nodal metastasis (*p* < 0.001, *p* = 0.001) and clinical stage (*p* < 0.001, *p* = 0.049) also correlated significantly with both DFS and OS. T-stage correlated significantly with DFS (*p* < 0.001), but not OS (*p* = 0.147). In the multivariate analysis, we included age at treatment, sex, subsite, clinical stage and MTV _(T, 2.5)_. With regard to DFS, MTV _(T, 2.5)_ (HR 6.97 [95% CI: 2.13–22.80], *p* = 0.001) and clinical stage (HR 4.32 [95% CI: 1.11–16.8], *p* = 0.034) were both found to be independent prognostic factors. With regard to OS, only MTV _(T, 2.5)_ was found to be an independent prognostic factor (HR 1.96 [95% CI: 0.63–3.11], *p* = 0.002).

**Table 2:** Comparison of disease free survival and overall survival to clinical factors (n = 118).

		No. of Patients (%)		Disease free survival (%)		Fisher *p* value	Overall survival (%)		Fisher *p* value
**Age**									
	< 69	60	(50.8%)	46	(76.7%)	0.832	54	(90.0%)	0.579
	≥ 69	58	(49.2%)	43	(74.1%)		50	(86.2%)	
**Gender**									
	male	115	(97.5%)	86	(74.8%)	1	102	(88.7%)	0.318
	female	3	(2.5%)	3	(100%)		2	(66.7%)	
**Subsite**									
	Glottic	84	(71.2%)	69	(82.1%)	0.01	78	(92.9%)	0.024
	Non glottic	34	(28.8%)	20	(58.8%)		26	(76.5%)	
**T Stage**									
	T1-T2	73	(61.9%)	67	(91.8%)	<0.001	67	(91.8%)	0.147
	T3-T4	45	(38.1%)	22	(48.9%)		37	(82.2%)	
**N Stage**									
	N(-)	90	(76.3%)	76	(84.4%)	<0.001	85	(94.4%)	0.001
	N(+)	28	(23.7%)	13	(46.4%)		19	(67.9%)	
**TMN Stage**									
	Stage I—II	64	(54.2%)	60	(93.7%)	<0.001	60	(93.8%)	0.049
	Stage III—IV	54	(45.8%)	29	(53.7%)		44	(81.5%)	
**MTV**_**(T, 2.5)**_									
	< 4.9 ml	81	(68.6%)	74	(91.4%)	<0.001	77	(95.1%)	0.001
	≥ 4.9 ml	37	(31.4%)	15	(40.5%)		27	(73.0%)	

Abbreviations: MTV = metabolic tumor volume.

**Table 3:** Multivariate analysis of clinical factors related to disease free survival and overall survival (n = 118).

	Disease free survival			Overall survival		
	HR	95% CI	*p* value	HR	95% CI	*p* value
**TMN Stage**						
Stage I—II	1					
Stage III—IV	4.32	1.11–16.8	0.034			
**MTV**_**(T, 2.5)**_						
< 4.9 ml	1			1		
≥ 4.9 ml	6.97	2.13–22.80	0.001	1.96	0.63–3.11	0.002

Abbreviations: HR = hazard ratio; CI = confidence interval; MTV = metabolic tumor volume.

**Fig 3:**
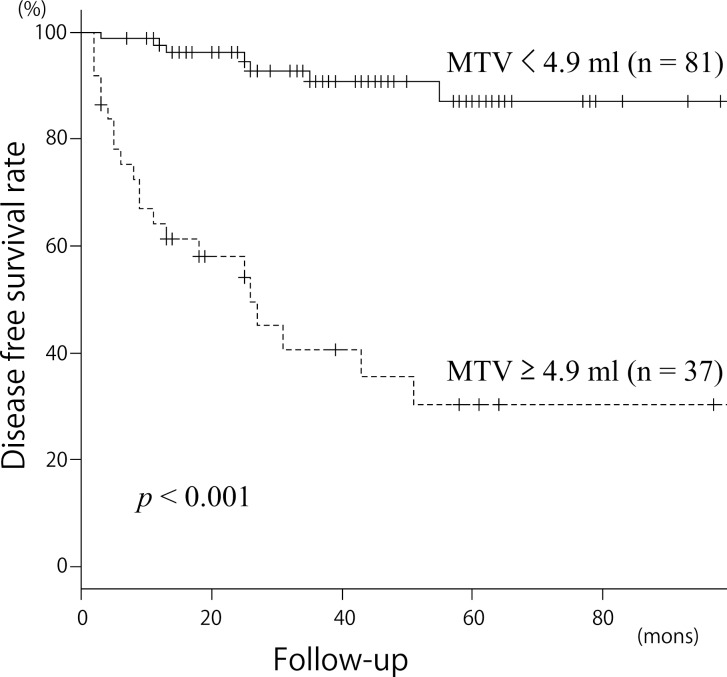
Kaplan-Meier curves for disease free survival according to MTV _(T, 2.5)_. *Upper line* MTV _(T, 2.5)_ < 4.9 ml (n = 81); *lower line* MTV _(T, 2.5)_ ≥ 4.9 ml (n = 37); *p* < 0.001 for locoregional control.

**Fig 4:**
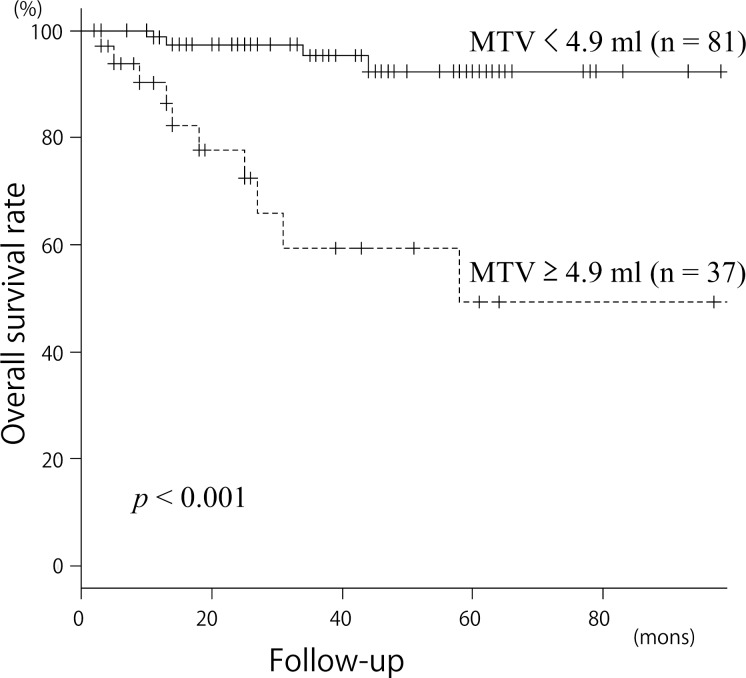
Kaplan-Meier curves for overall survival according to MTV _(T, 2.5)_. *Upper line* MTV _(T, 2.5)_ < 4.9 ml (n = 81); *lower line* MTV _(T, 2.5)_ ≥ 4.9 ml (n = 37); *p* < 0.001 for overall survival.

The Kaplan–Meier curves for DFS and OS in patients with an MTV _(T, 2.5)_ ≥ 4.9 ml or < 4.9 ml are presented in Figs. 3 and 4. The 3-year DFS for patients with an MTV _(T, 2.5)_ < 4.9 ml or ≥ 4.9 ml were 92.94% and 38.65%, respectively (*p* < 0.001). The 3-year OS for patients with an MTV _(T, 2.5)_ < 4.9 ml or ≥ 4.9 ml were 95.36% and 59.27%, respectively (*p* < 0.001). These results indicated that an MTV _(T, 2.5)_ ≥ 4.9 ml was a significant predictor of a poorer prognosis.

## Discussion

It is essential to evaluate tumor extent for clinical staging and the development of a treatment strategy in patients with laryngeal carcinoma. In concrete terms, confirmation of vocal cord fixation or infiltration to another subsite, evaluation of the contralateral vocal cord by laryngoscopy, and evaluation of invasion to cartilage, paraglottic space or extralaryngeal area by imaging modalities such as CT or MRI are all part of the mandatory work-up before considering a therapeutic plan. MTV, a volumetric parameter of PET, can be used to evaluate the tri-dimensional extent as well as the biological activity of a tumor. In this retrospective study, multivariate analysis revealed that, rather than clinical staging based on conventional modalities, pretreatment MTV measurement of laryngeal cancer was a prognostic factor for determining DFS. The risk of residual or recurrent disease after CCRT was approximately seven times higher in patients with a high MTV than in patients with a low MTV. The present study included patients with laryngeal cancer treated homogenously with irradiation or CCRT, resulting in a reduction in variations in the anatomical and clinical features of the cancer. Under this condition, a higher MTV indicated a poorer treatment outcome, suggesting that it might be used to select therapeutic strategy. In other words, patients with a higher MTV could be candidates for radical surgery, such as total laryngectomy in consideration of the poor prognosis for treatment with CCRT. Conversely, patients with a lower MTV might be candidates for less-aggressive treatments, such as irradiation alone or the concurrent use of less-toxic radiosensitizers.

Measurement of MTV requires a tri-dimensional image analysis system. The system we utilized was a commercial software program included in the electric medical chart system of our hospital and commonly used for CT angiography or the detailed examination of bone fractures or bronchial tube deformities. Delineation of the SUV threshold could be performed by a simple mouse-driven operation. Considering that the evaluation of nodal or distant-disease staging of head and neck carcinoma by PET or PET/CT has become widespread, additional evaluation of MTV results in little additional burden on the patients.

The current study used three SUV thresholds to delineate and two patterns of volume of interest; fixed SUV isocontours of 2.0, 2.5 and 3.0, and definition of the region as the tumor or the tumor plus metastatic lymph nodes. A higher SUV threshold resulted in a slightly wider AUC, which is expected to be more accurate. The fact that a higher SUV threshold can avoid physiological FDG uptake by the surrounding tissue may be a major reason for this result. On the other hand, the evaluation of MTV in small tumors is difficult when a higher SUV threshold is used. In our cohort, the number of patients with MTV _(T, 2.5)_ ≥ 4.9 ml and that of patients with MTV _(T, 3.0)_ ≥ 1.9 ml were almost same, and a fixed SUV of 2.5 was commonly adopted in former reports [4, 7, 8]. We, therefore, used MTV _(T, 2.5)_ in further investigations of prognostic value.

In the present study, we also evaluated the validity and predictive value of pretreatment MTV for RT or CCRT. Patients with a CR showed a significantly lower MTV than those with residual disease after primary treatment. The PPV for a CR after primary treatment in patients with a primary tumor with a low MTV (MTV _(T, 2.5)_ < 4.9 ml) was 0.98. The specificity and accuracy were 0.89 and 0.81, respectively. A high PPV suggests that there is a high probability that a primary tumor with a low MTV can be controlled by CCRT or RT. On the other hand, a tumor with a high MTV does not necessarily indicate treatment failure, as indicated by the low NPV. However, a tumor with a high MTV was 4.5 times more likely to relapse than one with a low MTV even after an initial CR was obtained (28.3% vs 6.3%).

The prognostic value of the MTV of the primary tumor plus metastatic lymph node is still controversial. Garsa et al. reported that the metabolic parameters of the primary tumor and all metabolically involved lymph nodes (if present) were of greater prognostic value than those of the primary tumor alone [5]. Tang et al. investigated primary tumor MTV and nodal MTV separately, and reported that primary tumor MTV could be used to predict progression-free and overall survival, but nodal MTV did not predict either [6]. In the present study, we measured tumor plus nodal MTV in 27 patients with lymph node metastasis. ROC estimation revealed that the accuracy of tumor plus nodal MTV was lower than that of tumor MTV. This is mainly due to the difference in radiosensitivity between the primary tumor and metastatic lymph nodes. Therefore, further studies are needed to elucidate the value of nodal MTV.

Potential limitations of this study include its single-institution retrospective design, non-uniform concurrent chemotherapy, uneven distribution of subsites and difference of spatial resolution between PET scanner and PET/CT device. Despite these limitations, this report is noteworthy because it is the first study to show the predictive value of MTV in patients with laryngeal cancers, and our findings suggest the need for further studies on the role of MTV in the selection of treatment modalities for this disease.

In conclusion, MTV of the primary tumor is a significant prognostic factor for DFS and OS in patients with laryngeal carcinoma treated by RT alone or CCRT. The results imply that MTV could be an important factor when planning treatment and follow-up for patients with laryngeal carcinoma. A prospective study is required to better clarify whether MTV is useful for optimizing the treatment strategy for laryngeal cancer.
